# Does contemporary vancomycin dosing achieve therapeutic targets in a heterogeneous clinical cohort of critically ill patients? Data from the multinational DALI study

**DOI:** 10.1186/cc13874

**Published:** 2014-05-15

**Authors:** Stijn Blot, Despoina Koulenti, Murat Akova, Matteo Bassetti, Jan J De Waele, George Dimopoulos, Kirsi-Maija Kaukonen, Claude Martin, Philippe Montravers, Jordi Rello, Andrew Rhodes, Therese Starr, Steven C Wallis, Jeffrey Lipman, Jason A Roberts

**Affiliations:** 1 Department of Internal Medicine, Faculty of Medicine & Health Science, Ghent University, Sint-Pietersnieuwstraat 25, 9000 Ghent, Belgium; 2 Burns Trauma and Critical Care Research Centre, The University of Queensland, Butterfield Street, Herston, Brisbane, QLD 4029, Australia; 3 Royal Brisbane and Women’s Hospital, Butterfield Street, Herston, Brisbane, QLD 4006, Australia; 4 Attikon University Hospital, 1, Rimini Street, Haidari, 124 62 Athens, Greece; 5 Hacettepe University, School of Medicine, 06100 Sıhhiye, Ankara, Turkey; 6 Azienda Ospedaliera Universitaria Santa Maria della Misericordia, Piazzale Santa Maria della Misericordia, 15, 33100 Udine, Italy; 7 Ghent University Hospital, De Pintelaan 185, 9000 Ghent, Belgium; 8 Helsinki University Central Hospital, Haartmaninkatu 3, 00029 Helsinki, Uusimaa, Finland; 9 Australian and New Zealand Intensive Care Research Centre (ANZIC RC), Department of Epidemiology and Preventive Medicine, Monash University, 99 Commercial Road, Melbourne, VIC 3004, Australia; 10 Hospital Nord, Chemin des Bourrely, 13015 Marseille, France; 11 AzuRea Group, France; 12 Centre Hospitalier Universitaire Bichat-Claude Bernard, AP-HP, Université Paris VII, 46 Rue Henri Huchard, 75018 Paris, France; 13 CIBERES, Vall d'Hebron Institute of Research, Universitat Autonoma de Barcelona, Passeig de la Vall d’Hebron, 119-129, 08035 Barcelona, Spain; 14 St George’s Healthcare NHS Trust and St George’s University of London, Cranmer Terrace, London SW17 0RE, UK

## Abstract

**Introduction:**

The objective of this study was to describe the pharmacokinetics of vancomycin in ICU patients and to examine whether contemporary antibiotic dosing results in concentrations that have been associated with favourable response.

**Methods:**

The Defining Antibiotic Levels in Intensive Care (DALI) study was a prospective, multicentre pharmacokinetic point-prevalence study. Antibiotic dosing was as per the treating clinician either by intermittent bolus or continuous infusion. Target trough concentration was defined as ≥15 mg/L and target pharmacodynamic index was defined as an area under the concentration-time curve over a 24-hour period divided by the minimum inhibitory concentration of the suspected bacteria (AUC_0–24_/MIC ratio) >400 (assuming MIC ≤1 mg/L).

**Results:**

Data of 42 patients from 26 ICUs were eligible for analysis. A total of 24 patients received vancomycin by continuous infusion (57%). Daily dosage of vancomycin was 27 mg/kg (interquartile range (IQR) 18 to 32), and not different between patients receiving intermittent or continuous infusion. Trough concentrations were highly variable (median 27, IQR 8 to 23 mg/L). Target trough concentrations were achieved in 57% of patients, but more frequently in patients receiving continuous infusion (71% versus 39%; *P* = 0.038). Also the target AUC_0–24_/MIC ratio was reached more frequently in patients receiving continuous infusion (88% versus 50%; *P* = 0.008). Multivariable logistic regression analysis with adjustment by the propensity score could not confirm continuous infusion as an independent predictor of an AUC_0–24_/MIC >400 (odds ratio (OR) 1.65, 95% confidence interval (CI) 0.2 to 12.0) or a C_min_ ≥15 mg/L (OR 1.8, 95% CI 0.4 to 8.5).

**Conclusions:**

This study demonstrated large interindividual variability in vancomycin pharmacokinetic and pharmacodynamic target attainment in ICU patients. These data suggests that a re-evaluation of current vancomycin dosing recommendations in critically ill patients is needed to more rapidly and consistently achieve sufficient vancomycin exposure.

## Introduction

Methicillin-resistant *Staphylococcus aureus* (MRSA) has become a major pathogen in severe healthcare-associated infections [1]. In the past decades, the anti-MRSA armamentarium has broadened. Still, vancomycin remains the most common first-line option for treating severe infections with parenteral therapy [2]. Achievement of pharmacokinetic/pharmacodynamic (PK/PD) indices associated with maximal bacterial kill is recommended to increase the likelihood of clinical cure. To achieve target serum concentrations in life-threatening infections such as sepsis, infective endocarditis, osteomyelitis and hospital-acquired pneumonia, current guidelines recommend trough serum concentrations of vancomycin (C_min_) ranging 15 to 20 mg/L [3]. Such serum concentrations should achieve an area under the concentration-time curve over a 24-hour period divided by the minimum inhibitory concentration of the suspected bacteria (AUC_0–24_/MIC ratio) of >400 (assuming an MIC ≤1 mg/L). This threshold was significantly associated with favourable clinical and bacteriological outcomes in patients with lower respiratory tract infections and therefore generally accepted as the appropriate PK/PD target [4]. Other investigators confirmed that an AUC_0–24_/MIC ratio for optimizing clinical outcomes should be at least 400 [5,6].

In critically ill patients with severe sepsis or septic shock however, target concentrations may be difficult to achieve due to the increased distribution volume and the presence of augmented renal clearance (ARC) [7-9]. These factors may lead to reduced trough concentrations and underdosing, leading to inadequate bacterial killing and possible treatment failure. Moreover, insufficient dosing may facilitate development of multidrug resistance. As vancomycin is renally cleared and distributes widely throughout the body, its pharmacokinetics may be altered from the pathophysiological alterations inherent to sepsis. Furthermore, these factors imply great interindividual variability in pharmacokinetics complicating accurate prediction of serum concentrations in septic patients.

Alternatively, the course of ICU patients may be complicated by acute kidney injury (AKI), in which case decreased vancomycin clearance and subsequent increased trough concentrations may cause toxicity. Should AKI worsen, renal replacement therapy (RRT) may be required with drug clearance varying according to mode of RRT, dose of RRT delivered, filter material and surface area, and blood flow rate [10,11]. As such, factors that may lead to underdosing as well as overdosing are to be considered. The above-mentioned conditions call for an altered dosing approach in critically ill patients compared with mild-to-moderately ill patients [12-14]. Several investigators reported on pharmacokinetics of vancomycin in critically ill patients [5,6,15,16] but most studies are single centre designs and there is no insight in current practice - and therefore in pharmacokinetics variability - across ICUs.

The objective of this study was to describe the pharmacokinetics of vancomycin in critically ill patients and to examine whether contemporary antibiotic dosing for ICU patients achieves concentrations that are associated with favourable response in previous reports. We hypothesised that vancomycin target trough concentrations and AUC_0–24_/MIC are frequently not achieved and demonstrate considerable variability in critically ill patients. Furthermore, we investigated the role of continuous infusion of vancomycin. We also studied factors associated with favourable pharmacokinetic outcome (trough concentration ≥15 mg/L and AUC_0–24_/MIC ratio >400).

## Methods

### Study design

The Defining Antibiotic Levels in Intensive Care (DALI) study was a prospective, multicentre pharmacokinetic point-prevalence study. For detailed information on methods we refer the reader to the protocol of DALI study that has been previously published [17]. In this secondary study patients receiving intravenous vancomycin, intermittently or continuously, were analysed.

### Patients

Critically ill adult patients were all identified for participation by clinical ICU staff on the Monday of the nominated sampling week, with blood sampling and data collection occurring throughout that week. Ethical approval to participate in this study was obtained at all participating centres and informed consent was obtained from each patient or their legally authorised representative. The lead site was The University of Queensland, Australia with ethical approval granted by the Medical Research Ethics Committee (no. 201100283, May 2011). The contributing authors are listed in Additional file 1 and the sites and their ethical approval bodies are listed in Additional file 2.

### Intervention

Antibiotic dosing was as per the treating clinician and therapy could be administered by either intravenous intermittent or continuous infusion. Each patient had two blood samples taken: for intermittent administration of vancomycin, blood sample A was a mid-dose blood sample at 50% of the way through a dosing interval and blood sample B was a pre-dose concentration at the end of a dosing interval (within 30 minutes of the next dose), while for continuous administration sample A was at any time and sample B was >6 hours after sample A. The observed concentrations were then interpreted in relation to a presumed MIC of 1 mg/L.

### Data collection

Data collection was performed by trained staff at each participating centre and entered onto a case report form (CRF). Various demographic and clinical data were collected, including age, gender, height, weight, admission diagnosis, presence of extracorporeal circuits (for example RRT), clinical outcome of infection, presence/absence of surgery within previous 24 hours, and mortality at 30 days. Also, organ function data (including renal function - serum creatinine concentration during studied dosing interval; eight-hour urinary creatinine clearance (where available), fluid balance for total length of stay and previous 24 hours), antibiotic dosing (dose and frequency, time of dosing and sampling, day of antibiotic therapy), and infection data (including known or presumed pathogen) were collected.

Antibiotic dosing data including the dose, infusion duration, frequency of administration, the time of dosing and sampling and the day of antibiotic therapy were collected. All data were collated by the coordinating centre (Burns, Trauma and Critical Care Research Centre, The University of Queensland, Australia).

### Bioanalysis

Blood samples were processed and stored per protocol to maintain integrity. Vancomycin concentrations in serum were determined by a validated chromatographic assay method on an Applied Biosystems API2000 mass spectrometer (Applied Biosystems, Foster City, CA, USA) with Shimadzu high-performance liquid chromatography (HPLC) system (Shimadzu Corp., Kyoto, Japan). The stationary phase was a Waters X-Terra C18 column (5 μm, 150 × 2.1 mm) (Waters Corp., Milford, MA, USA) and the mobile phase was a gradient (between 20% and 95% organic) of methanol containing 10 mM heptafluorobutyric acid (HFBA) with 0.1% formic acid containing 10 mM HFBA. Vancomycin and the internal standard (tobramycin) were detected using multiple reaction monitoring (MRM) scans in positive ion detection mode with electrospray ionisation (ESI) sample introduction. Vancomycin standards and quality controls were prepared in serum. One hundred microlitres of sample was mixed with the internal standard and the sample then de-proteinated with acetonitrile and lipids removed with dichloromethane before injection. The method fitted a quadratic regression profile from 0.1 to 100 μg/mL. Accuracy and precision were determined from quality controls at high, medium and low concentrations and were within 10% at all levels. These methods are in accordance with the US Food and Drug Administration’s guidance for industry on bioanalysis [18].

### Pharmacokinetic analyses

The pharmacokinetic values were calculated using non-compartmental methods. The apparent terminal elimination rate constant (kel) was determined from log-linear least squares regression analysis of concentrations from sample A and B. For intermittent infusions, the maximum concentration for the dosing period (C_max_) was calculated using the following equation:

$$C_{\text{max}}=C_{0}/\left(1–{e^{-\mathrm{kel}.}}^{\tau }\right)$$

Where C_0_ is the concentration at sample A and τ is the dosing interval in hours. The minimum concentration for the dosing period (C_min_) was the observed value. Half-life (T_1/2_) was calculated as ln(2)/_z_. The area under the concentration time curve from 0 to 12 hours (AUC_0–12_) was calculated using the linear trapezoidal rule with the derived C_max_ estimate. The AUC from 0 to 24 hours (AUC_0–24_) was calculated using a doubling of AUC_0–12_ with pharmacokinetic steady-state assumed for all patients. For patients receiving continuous infusion, the AUC_0–24_ was calculated using the average of concentrations for sample A and B multiplied by 24 hours. Vancomycin clearance (CL) was calculated using the equation dose/AUC_0–12_.

The AUC_0–24_/MIC ratio was calculated by dividing the AUC_0–24_ by MICs 0.5, 1 and 2 mg/L.

### Outcome variables

Vancomycin trough concentrations and AUC_0–24_/MIC ratios are the main PK/PD variables. The target trough concentration is defined as ≥15 mg/L and the target AUC_0–24_/MIC ratio is defined as >400.

### Statistical analyses

Basic statistics on demographic, clinical and PK/PD related data were presented by number (%), median [1^st^-3^rd^ quartile] or mean (standard deviation (SD)) as appropriate. Chi-square and Mann-Whitney *U* test were used as appropriate. Logistic regression analysis was used to assess unadjusted associations with the target PD index (AUC_0–24_/MIC ratio >400) or target trough concentration (≥15 mg/L) and independent variables. Various clinical and demographic factors were evaluated for entering into the model. Multivariable logistic regression was used to assess relationships between continuous infusion of vancomycin and the target PD index. In these analyses adjustment for covariates was obtained by means of a propensity score estimated for each subject on basis of a logistic regression of treatment (continuous or intermittent infusion) received on covariates. The propensity score represents a single variable reflecting the effect of the covariants on the probability of receiving continuous infusion. It summarises covariates with a statistical or plausible relationship with continuous infusion therapy or serum concentrations. Covariates used in the final propensity model were age, gender, sequential organ failure assessment (SOFA) score, RRT, fluid balance, vancomycin dose (mg/kg/24 h), infection source, and number of days on therapy. The area under the curve, representing the probability that the patient would receive continuous infusion based on the variables included, was 0.82 (95% confidence interval (CI) 0.68 to 0.96). Results of the regression analyses are reported as odds ratios (OR) and 95% CI. The Hosmer-Lemeshow test was used to assess whether or not the observed event rates match expected event rates in subgroups of the model population. Statistical significance was defined as *P* <0.05.

## Results

Vancomycin blood samples were collected from 45 critically ill patients. Three patients were excluded as their samples were not viable. As such, the study sample consisted of 42 study subjects from 26 ICUs of eight countries (Andorra, Belgium, Spain, UK, Greece, Italy, Portugal, Turkey). Demographic data, disease severity, and renal function are described in Table 1. Data are presented for patients receiving intermittent versus continuous vancomycin therapy. Vancomycin was given empirically in 31 patients (73.8%). Therapy was targeted in only 11 patients. MICs for isolated *Staphylococcus* spp. were only reported in two cases (both MIC 1 mg/L).

**Table 1:** **Characteristics of 42 critically ill patients receiving vancomycin therapy**, **stratified for intermittent dosing or continuous infusion**

**Characteristic**	**All patients (n = 42)**	**Intermittent dosing (n = 18)**	**Continuous infusion (n = 24)**	P^*^
Age (years)	58 [45 **-** 66]	58 [32 **-** 79]	58 [45 **-** 66]	0.755
Male sex	27 (64.3)	13 (72.2)	14 (58.3)	0.353
Weight (kg)	70 [61 **-** 85]	75 [65 **-** 88]	70 [61 **-** 85]	0.350
APACHE II score	16 [13 **-** 25]	17 [14 **-** 27]	16 [13 **-** 25]	0.920
SOFA score	6 [3 **-** 9]	5 [3 **-** 6]	6 [3 **-** 9]	0.595
Serum creatinine concentration (μmol/L)	65 [51 **-** 113]	64 [52 **-** 91]	74 [44 **-** 129]	0.751
Urinary creatinine clearance (mL/min.)	85 [60 **-** 106]	72 [20 **-** 90]	101 [74 **-** 167]	0.198
Renal replacement therapy	9 (21.4)	1 (5.6)	8 (33.3)	NA
Intermittent hemodialysis	2 (4.8)	0	2	
Continuous renal replacement therapy	7 (16.7)	1	6	

Data are described as median [1^st^ - 3^rd^ quartile] or n (%). ^*^Indicates difference between intermittent dosing and continuous infusion of vancomycin. APACHE II, acute physiology and chronic health evaluation II; SOFA, sequential organ failure assessment; NA, not applicable as the expected count in at least one cell is less than 5.

**Figure 1:**
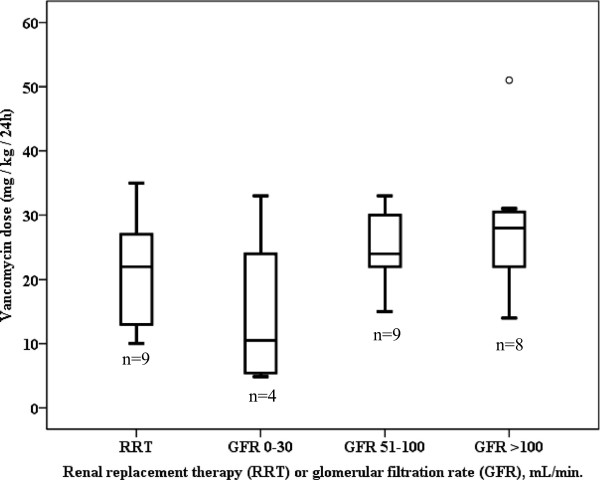
Boxplots of vancomycin dosages administered stratified for renal replacement therapy and various glomerular filtration rates. ^*^Data on glomerular filtration were only available in 30 patients. No difference between groups could be demonstrated (*P* = 0.359).

Data on dosing, pharmacokinetics and PK/PD are reported in Table 2. Twenty-four patients received vancomycin by continuous infusion (57.1%). The average daily dosage of vancomycin was 27 (SD 13) mg/kg and was not different between patients in the continuous infusion and intermittent dosing group. No difference was observed in daily dosing between patients receiving RRT or with different glomerular filtration rates (GFRs) (Figure 1) (*P* = 0.359).

**Table 2:** Pharmacokinetic parameters and PK/PD target attainment of vancomycin in critically ill patients

**Parameter**	**All patients (n = 42)**	**Intermittent dosing (n = 18)**	**Continuous infusion (n = 24)**	P^*^
**Vancomycin dose (mg/kg)**	27 [18 - 32]	27 [22 - 30]	27 [17 - 33]	0.611
**Elimination rate constant (h**^**-1**^)**	0.09 [0.03 - 0.13]	0.09 [0.03 - 0.13]	-	-
**Clearance (L/h)**	3.6 [1.9 - 5.9]	5.1 [2.4 - 7.1]	2.7 [1.7 - 4.1]	0.038
Half-life (h)^**^	8.2 [5.4 - 24.1]	8.2 [5.4 - 24.1]	-	-
**C**_**min **_**(mg/L)**	17 [8 - 23]	10 [7- 17]	21 [14 - 26]	0.029
**C**_**min **_**≥15 mg/L, n (percentage)**	24 (57.1)	7 (38.9)	17 (70.8)	0.038
**AUC**_**0–24**_**/MIC**	655 [368 - 911]	409 [246 - 712]	830 [529 - 952]	0.029
**AUC**_**0–24**_**/MIC > 400, n (percentage)**	30 (71.4)	9 (50.0)	21 (87.5)	0.008
**Length of vancomycin therapy on sampling date**				
**Days, n**	4 [1 - 7]	2 [1 - 6]	4 [1 - 7]	0.314
**>2 days, n (percentage)**	23/41 (56.1)	8/18 (44.4)	15/23 (65.2)	0.183
**>3 days, n (percentage)**	22/41 (53.7)	8/18 (44.4)	14/23 (60.9)	0.295

Data are described as median [1^st^ - 3^rd^ quartile] or n (%). For pharmacodynamics calculations, an MIC of 1 mg/L was assumed. ^*^Indicates difference between intermittent dosing and continuous infusion of vancomycin; ^**^could only be determined in patients receiving intermittent dosing. C_min_, minimum concentration of drug observed during the dosing period; AUC_0–24_/MIC, ratio of the area under the concentration-time curve over a 24-hour period divided by the minimum inhibitory concentration of the suspected bacteria.

**Figure 2:**
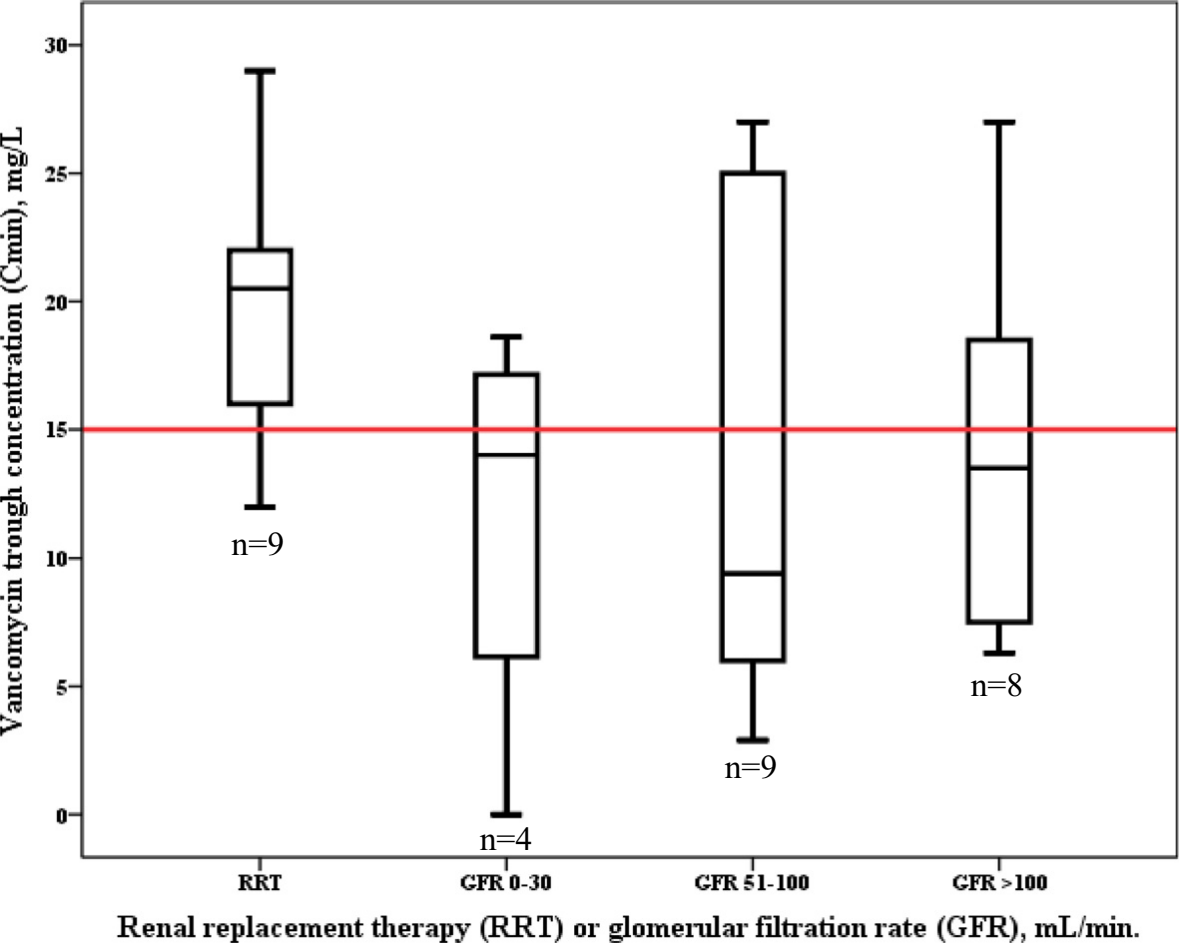
Boxplot of vancomycin trough concentrations stratified for renal replacement therapy and various glomerular filtration rates. ^*^Data on glomerular filtration were only available in 30 patients. Red line indicates vancomycin trough concentration target (15 mg/L). No difference between groups could be demonstrated (*P* = 0.233).

**Figure 3:**
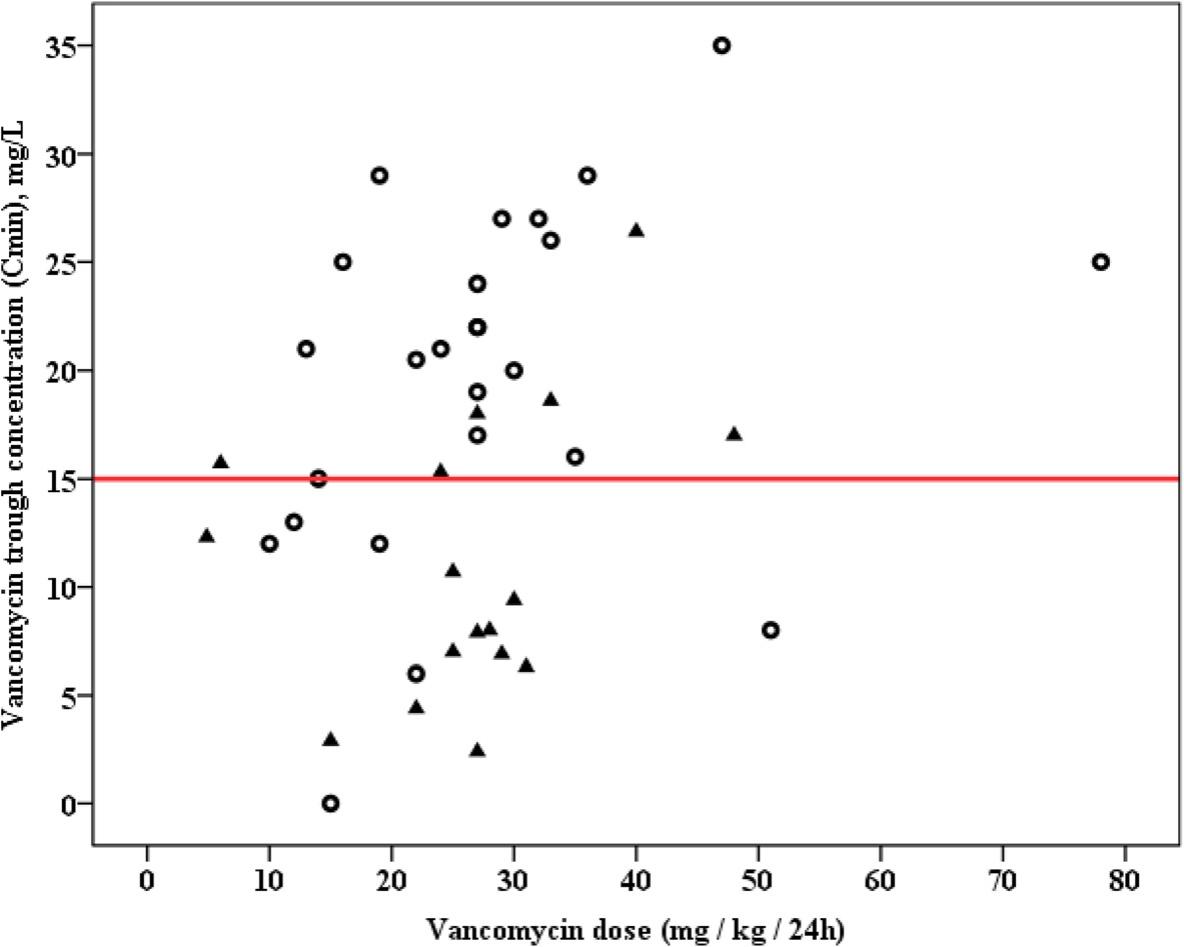
Vancomycin trough concentration according to daily dose administered. ^*^Red line indicates target trough concentration of 15 mg/L; circles represent patients with continuous infusion; triangles represent patients with intermittent dosing. Spearman rank correlation for (i) all patients: R = 0.540 (*P* <0.001), (ii) patients with continuous infusion: R = 0.587 (*P* = 0.062), and patients with intermittent dosing: R = 0.127 (*P* = 0.615).

Target trough concentrations (≥15 mg/L) were achieved in 57% of patients (Table 2). Trough concentrations did not differ between patients with either RRT or various degrees of glomerular filtration (Figure 2) (*P* = 0.233), but were more frequently achieved with continuous infusion therapy (Table 2). Figure 3 illustrates trough concentrations according to weight-adjusted daily doses administered and stratified for continuous or intermittent dosing.

**Figure 4:**
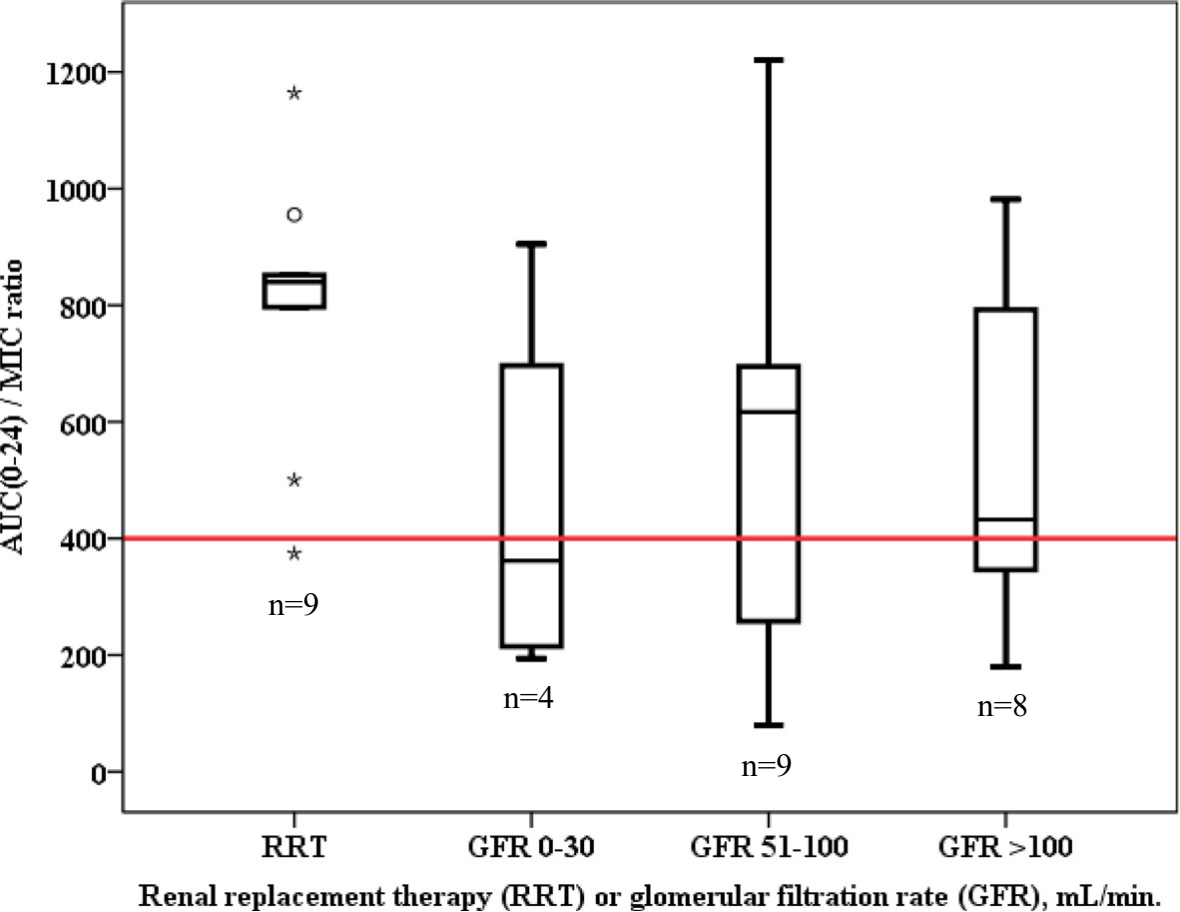
**Boxplot of vancomycin AUC**_**0–24**_/MIC ratios stratified for renal replacement therapy and various glomerular filtration rates. ^*^Data on glomerular filtration were only available in 30 patients. Red line indicates the AUC_0–24_/MIC target ratio (400). No difference between the groups could be demonstrated (*P* = 0.224). AUC_0–24_/MIC, ratio of the area under the concentration-time curve over a 24-hour period divided by the minimum inhibitory concentration of the suspected bacteria.

**Figure 5:**
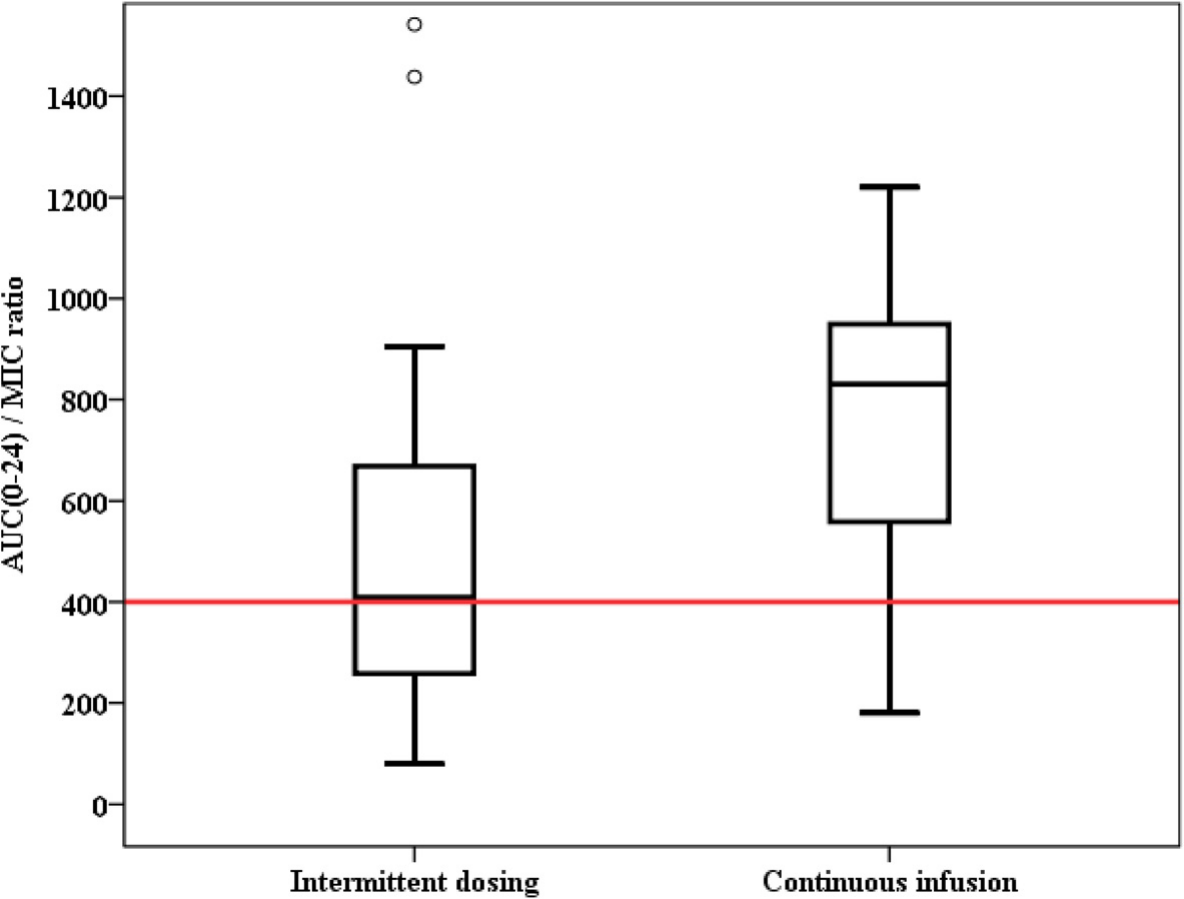
**AUC**_**0**–**24**_/MIC ratios of vancomycin for intermittent dosing versus continuous infusion. ^*^Boxes indicate lower quartile (bottom of box), median (band near the middle of the box), and upper quartile (top of the box); whiskers indicate the lowest value still within 1.5 of the interquartile range from the lower quartile, and the highest value still within 1.5 of the interquartile range from the upper quartile; circles indicate outliers. The red line indicates the minimum the target threshold for optimizing outcomes (AUC_0–24_/MIC >400). Difference between the groups: *P* = 0.029. AUC_0–24_/MIC, ratio of the area under the concentration-time curve over a 24-hour period divided by the minimum inhibitory concentration of the suspected bacteria.

The target AUC_0–24_/MIC (>400) was reached in 71% of the study subjects if an MIC = 1 mg/L was assumed. If MIC values of 0.5 or 2 mg/L were used, then the AUC_0–24_/MIC (>400) target would have been reached in 88% and 38% of patients, respectively. AUC_0–24_/MIC ratios were not statistically significantly different between patients with RRT or distinct degrees of glomerular filtration (Figure 4) (*P* = 0.224). The target AUC_0–24_/MIC ratio was more frequently achieved with continuous infusion therapy (Figure 5) (*P* = 0.008).

Table 3 describes univariate relationships between covariates and PD target values as assessed by logistic regression analyses. Multivariable logistic regression analysis with adjustment by the propensity score could not confirm continuous infusion as an independent predictor of an AUC_0–24_/MIC >400 (OR 1.65, 95% CI 0.2 to 12.0) or a C_min_ ≥15 mg/L (OR 1.8, 95% CI 0.4 to 8.5). Hosmer and Lemeshow tests for both models were respectively *P* = 0.660 and *P* = 0.506.

**Table 3:** **Unadjusted relationships with AUC**_**0–24**_**/MIC ratio >400 and target trough concentrations (>15 mg/L)**

**Variable**	**Unadjusted relationships with AUC**_**0–24**_**/MIC ratio >400**		**Unadjusted relationships with C**_**min **_**>15 mg/L**	
	**OR (95% CI)**	***P***	**OR (95% CI)**	***P***
**Age (/year increase)**	0.99 (0.95 - 1.03)	0.547	0.99 (0.96 - 1.03)	0.723
**Male sex**	0.59 (0.13 - 2.69)	0.499	1.20 (0.33 - 4.32)	0.780
**SOFA score (/point increase)**	1.10 (0.91 - 1.32)	0.318	1.05 (0.91 - 1.21)	0.519
**Renal replacement therapy**	3.64 (0.40 - 33.12)	0.252	3.29 (0.59 - 18.3)	0.173
**Fluid balance of the latest 24 hours**				
**Negative fluid balance**	2.20 (0.49 - 9.88)	0.305	1.12 (0.32 - 3.91)	0.856
**Negative fluid balance >1000 mL**	2.40 (0.26 - 22.55)	0.444	1.00 (0.19 - 5.15)	0.999
**Positive fluid balance**	0.46 (0.10 - 2.05)	0.305	0.89 (0.26 - 3.10)	0.856
**Positive fluid balance >1000 mL**	0.46 (0.11 - 1.85)	0.273	0.75 (0.22 - 2.60)	0.650
**Positive fluid balance >2000 mL**	0.23 (0.05 - 1.06)	0.059	0.68 (0.16 - 2.85)	0.602
**Serum creatinine, mg/dL (/mg increase)**	1.02 (0.71 - 1.48)	0.914	2.71 (0.78 - 9.37)	0.116
**Creatinine clearance, mL/min. (/mL increase)**	1.02 (0.99 - 1.04)	0.194	1.00 (0.98 - 1.01	0.457
**Infection source pneumonia or intra-abdominal infection**	2.84 (0.52 - 15.47)	0.227	4.2 (0.97 - 18.53)	0.056
**Vancomycin dose, mg/kg (/mg increase)**	1.03 (0.97 - 1.10)	0.319	1.06 (0.99 - 1.14)	0.071
**Continuous infusion of vancomycin**	11.00 (1.98 - 61.26)	0.006	3.82 (1.05 - 13.91)	0.042
**Length of vancomycin therapy at sampling date**				
**Days (/day increase)**	1.35 (1.02 - 1.79)	0.034	0.94 (0.82 - 1.08)	0.370
**>2 days**	10.50 (1.88 - 58.62)	0.007	1.56 (0.45 - 5.41)	0.487
**>3 days**	9.00 (1.63 - 49.76)	0.012	1.30 (0.38 - 4.49)	0.687

AUC_0–24_/MIC, ratio of the area under the concentration-time curve over a 24-hour period divided by the minimum inhibitory concentration of the suspected bacteria; C_min_, minimum concentration of drug observed during the dosing period; OR, odds ratio; CI, confidence interval; SOFA, sequential organ failure assessment.

## Discussion

This is the first large-scale multicentre point-prevalence study investigating pharmacokinetics and PK/PD target attainment of vancomycin in critically ill patients. The present study revealed that a substantial proportion of patients did not achieve the target vancomycin exposures. Forty-five percent of patients did not reach the minimum C_min_ threshold (≥15 mg/L) while 26% did not reach an AUC_0–24_/MIC ratio >400. The latter index is generally considered as the most important factor for enhancing effectiveness of vancomycin. Several investigators have demonstrated that an AUC_0–24_/MIC ratio above 400 is required to optimise clinical outcomes [4-6]. It has been proposed that even higher index of exposure might be necessary in critically ill patients. In a cohort of patients with MRSA-associated septic shock, Zelenitsky *et al.* identified a threshold of AUC_0–24_/MIC >578 to be associated with higher likelihood of survival, while an AUC_0–24_/MIC <451 was associated with increased odds of death [15]. A threshold AUC_0–24_/MIC >578 is substantially higher than the standard cut-off ratio >400. In the present study only 24 patients (57%) reached an AUC_0–24_/MIC >578.

Another important observation from this study is the high variability in pharmacokinetics parameters. Wide ranges in C_min_, AUC_0–24_, and half-life were observed, and illustrate the challenge of vancomycin dosing in critically ill patients in comparison with mild-to-moderately sick patients in which pharmacokinetics is much more stable and, as such, predictable [19]. High interpatient variability in pharmacokinetics of antibiotics in critically ill patients has been reported previously [20-22]. Standard doses of the concentration-dependent agents amikacin and ciprofloxacin proved inadequate to achieve target peak concentrations in ICU patients [20,21]. Also standard dosing approaches for beta-lactam antibiotics appeared to be insufficient to achieve target concentrations in the early phase of sepsis [22]. Despite the generally accepted use of therapeutic drug monitoring (TDM) in vancomycin therapy and the administration of a wide range of doses in the present study, dosing was frequently suboptimal. Besides TDM data, dosing should be based on patient’s weight, and renal function. The average daily vancomycin doses administered for patients receiving RRT and patients with a GFR of 51 to 100 mL/min. or >100 mL/min., ranged from 20 to 30 mg/kg (Figure 1), and are therefore in accordance with recommendations. Notwithstanding this, more than half of the patients with GFR 51 to 100 or GFR >100 mL/min. did not achieve the target trough concentration of ≥15 mg/L (Figure 2). Particularly in the patient group with GFR 51 to 100 mL/min. adequate dosing seems to be problematic with a median C_min_ of 9 mg/L and high variability despite reasonable dosages administered. In patients with more impaired renal function (GFR 0 to 30 mL/min.) doses were restricted (median 10 mg/kg), also leading to inadequate trough concentrations in half of the patients. Patients receiving RRT most frequently achieved satisfying trough concentrations.

In the present study, continuous infusion of vancomycin appeared more successful for reaching target PD parameters than intermittent dosing (Tables 2 and 3). However, logistic regression failed to confirm this relationship. Continuous infusion of vancomycin in critically ill patients has been advocated before [23,24]. Although not recommended by the guidelines for the treatment of *S. aureus* infections or vancomycin therapy [2,3], the agent appears to be increasingly administered by continuous infusion. The present data cannot underscore the value of continuous infusion therapy for more consistently achieving PK/PD targets in this group of patients. Yet, previous reports stressed the potential value of continuously infused vancomycin stating that it may result in faster achievement of target concentrations [25], as well as more stable drug concentrations [26,27]. Although it seems reasonable to assume more favourable outcomes when PK/PD is optimised, clinical outcome data favouring continuous infusion are far from conclusive. In a cohort of patients with ventilator-associated pneumonia, Rello *et al.* found lower mortality rates in patients receiving vancomycin by continuous infusion (25% vs*.* 55%; *P* = 0.03) [28]. Although this finding was confirmed by logistic regression analysis, it should be interpreted cautiously as the study was primarily not designed for this purpose [24].

This point-prevalence study shows that vancomycin dosing practice results in too many patients being underdosed even with the availability of TDM. In order to overcome the risk of inadequate concentrations, new approaches should be considered such as the use of weight-based loading doses [13] as well as administration by continuous infusion. In one study by De Waele *et al.*, administering a loading dose of 1000 mg (<65 kg) or 1500 mg (>65 kg) followed by a continuous infusion of 2000 mg/24 h, the authors found that 78% and 88% of critically ill patients reached a serum concentration ≥15 mg/L on day two and three respectively [29]. In a dosing simulation analysis, Roberts *et al.* demonstrated that at least 35 mg/kg/24 h administered by continuous infusion is needed to maintain a concentrations ≥20 mg/L in some patients [13]. Alternatively, some authors have proposed the use of a vancomycin dosing nomogram for ICU patients. One recent nomogram that takes into account body weight for loading and maintenance dosing, and the Modification of Diet in Renal Disease equation for dosing frequency [30]. Although the nomogram resulted in a significant increase in patients achieving the target trough concentration, still 28% of patients had sub-therapeutic vancomycin exposures. Given the high variability in vancomycin concentrations, TDM should be considered essential, but as shown from this data, does not guarantee achievement of target concentrations.

This study has some limitations. First, we could not evaluate clinical outcome data as only nine patients had documented Gram-positive infections, the remaining patients were administered empiric therapy. However, this data does provide good insight into the PK/PD exposures of vancomycin that occur with contemporary dosing approaches. Second, PD calculations used assumed MIC values. For detailed analyses an assumed MIC = 1 mg/L was used because in Europe only 8% of *S. aureus* MICs for vancomycin are >1 mg/L [31]. Third, no data about potential loading doses were available.

## Conclusions

In conclusion, this study demonstrated large variability in vancomycin pharmacokinetics and PK/PD target attainment in critically ill patients. Continuous infusion was associated with higher likelihood to achieving clinically relevant trough concentrations (of ≥15 mg/L) pharmacodynamic exposure (AUC_0–24_/MIC ratio >400) in univariate analyses but not in multivariate analyses. These data support a re-evaluation of vancomycin dosing recommendations in critically ill patients with new approaches to more rapidly and consistently achieve clinically relevant PK/PD targets.

## Key messages

• We analysed vancomycin concentrations from 42 patients across 26 ICUs in eight countries. The indication for vancomycin was empiric (not directed) in 74% of cases.

• A total of 57% of patients were administered vancomycin by continuous infusion, although there was no difference in dose with patients administered intermittent dosing (27 mg/kg/day).

• Only 57% of patients achieved the target concentration (≥15 mg/L) and only 71% achieved a target AUC_0–24_/MIC of 400 (assuming a MIC of 1 mg/L).

• Although univariate analysis showed therapeutic targets were more consistently achieved with continuous infusion compared with intermittent dosing (*P* = 0.008), in multivariable logistic regression with adjustment by propensity score, this statistical significance was not maintained.

## Abbreviations

AKI: acute kidney injury; APACHE II: acute physiology and chronic health evaluation II score; ARC: augmented renal clearance; AUC_0–12_: area under the concentration time curve from 0 to 12 hours; AUC_0–24_/MIC: ratio of the area under the concentration-time curve over a 24-hour period divided by the minimum inhibitory concentration of the suspected bacteria; CI: confidence interval; CL: clearance; C_max_: maximum concentration of drug observed during the dosing period; C_min_: minimum concentration of drug observed during the dosing period; CRF: case report form; DALI: Defining Antibiotic Levels in Intensive Care; ESI: electrospray ionization; GFR: glomerular filtration rate; HFBA: heptafluorobutyric acid; HPLC: high-performance liquid chromatography; ICU: intensive care unit; kel: apparent terminal elimination rate constant; MIC: minimum inhibitory concentration of the suspected bacteria; MRM: multiple reaction monitoring; MRSA: methicillin-resistant *Staphylococcus aureus*; OR: odds ratio; PK: pharmacokinetics; PK/PD: pharmacokinetic/pharmacodynamic; RRT: renal replacement therapy; SD: standard deviation; SOFA: sequential organ failure assessment score; T_1/2_: half-life; TDM: therapeutic drug monitoring.

## Competing interests

The authors declare that they have no competing interests.

## Authors’ contributions

SB contributed to the analysis, interpretation, manuscript writing and final approval of the manuscript; DK contributed to the conception and design, data collection, analysis, interpretation, manuscript writing and final approval of the manuscript; MA contributed to the data collection, analysis, interpretation, manuscript writing and final approval of the manuscript; MB contributed to the data collection, analysis, interpretation, manuscript writing and final approval of the manuscript; JJD contributed to the conception and design, data collection, interpretation, manuscript writing and final approval of the manuscript; GD contributed to the conception and design, data collection, interpretation, manuscript writing and final approval of the manuscript; KMK contributed to the data collection, interpretation, manuscript writing and final approval of the manuscript; CM contributed to the conception and design, data collection, interpretation, manuscript writing and final approval of the manuscript; PM contributed to the conception and design, data collection, interpretation, manuscript writing and final approval of the manuscript; JR contributed to the conception and design, data collection, interpretation, manuscript writing and final approval of the manuscript; AR contributed to the conception and design, data collection, interpretation, manuscript writing and final approval of the manuscript; TS contributed to the conception and design, data collation, manuscript writing and final approval of the manuscript; SW contributed to the conception and design, analysis, interpretation, manuscript writing and final approval of the manuscript; JL contributed to the conception and design, interpretation, manuscript writing and final approval of the manuscript; JAR contributed to the conception and design, analysis, interpretation, manuscript writing and final approval of the manuscript. All authors read and approved the final manuscript.

## Supplementary Material

Additional file 1 A list of the DALI study authors. Click here for file

Additional file 2 A list of the contributing sites and their ethical approval bodies. Click here for file
