# Supplementary material for: Does contemporary vancomycin dosing achieve therapeutic targets in a heterogeneous clinical cohort of critically ill patients? Data from the multinational DALI study
Source: Crit Care. 2014 May 15;18(3):R99. doi: 10.1186/cc13874 (PMC4075416; doi:10.1186/cc13874)
Supplement: Additional file 1 — A list of the DALI study authors. [file cc13874-S1.docx]

**Appendix 1: DALI Study Authors**

| **Author name** | **Affiliation** |
| --- | --- |
| Jason A Roberts | Burns Trauma and Critical Care Research Centre, The University of Queensland, Brisbane, Australia;  Royal Brisbane and Women’s Hospital, Brisbane, Australia |
| Jeffrey Lipman | Burns Trauma and Critical Care Research Centre, The University of Queensland, Brisbane, Australia;  Royal Brisbane and Women’s Hospital, Brisbane, Australia |
| Therese Starr | Royal Brisbane and Women’s Hospital, Brisbane, Australia |
| Steven C Wallis | Burns Trauma and Critical Care Research Centre, The University of Queensland, Brisbane, Australia |
| Antonio Margarit Ribas | Hospital Nostra Senyora de Meritxell, Escaldes-Engordany, Andorra |
| Jan J. De Waele | Ghent University Hospital, Ghent, Belgium |
| Luc De Crop | Ghent University Hospital, Ghent, Belgium |
| Herbert Spapen | Universitair Ziekenhuis Brussels, Brussels, Belgium |
| Joost Wauters | Universitair Ziekenhuis Gasthuisberg, Leuven, Brussels |
| Thierry Dugernier | Clinique Saint Pierre, Ottignies, Belgium |
| Philippe Jorens | Universitair Ziekenhuis Antwerpen, Edegem, Belgium |
| Ilse Dapper | Algemeen Ziekenhuis Monica, Deurne, Belgium |
| Daniel De Backer | Erasme University Hospital, Brussels, Belgium |
| Fabio S. Taccone | Erasme University Hospital, Brussels, Belgium |
| Jordi Rello | Vall d'Hebron Institut of Research. Universitat Autonoma de Barcelona, Spain Centro de Investigación  Biomedica En Red- Enfermedades Respiratorias (CibeRes) |
| Laura Ruano | Vall d'Hebron Institut of Research. Universitat Autonoma de Barcelona, Spain Centro de Investigación  Biomedica En Red- Enfermedades Respiratorias (CibeRes) |
| Elsa Afonso | Hospital Universitari Vall d'Hebron. Vall d'Hebron Institut of Research. Universitat Autonoma de  Barcelona, Spain Centro de Investigación Biomedica En Red- Enfermedades Respiratorias (CibeRes) |
| Francisco Alvarez-Lerma | Hospital Del Mar, Parc Salut Mar. Barcelona, Spain |
| Maria Pilar Gracia-Arnillas | Hospital Del Mar, Parc Salut Mar. Barcelona, Spain |
| Francisco Fernández | Centro Médico Delfos, Barcelona |
| Neus Feijoo | Hospital General de L’Hospitalet, Barcelona, Spain |
| Neus Bardolet | Hospital General de L’Hospitalet, Barcelona, Spain |
| Assumpta Rovira | Hospital General de L’Hospitalet, Barcelona, Spain |
| Pau Garro | Hospital General de Granollers, Barcelona, Spain |
| Diana Colon | Hospital General de Granollers, Barcelona, Spain |
| Carlos Castillo | Hospital Txagorritxu, Vitoria, Spain |
| Juan Fernado | Hospital Txagorritxu, Vitoria, Spain |
| Maria Jesus Lopez | Hospital Universitario de Burgos. Burgos, Spain |
| Jose Luis Fernandez | Hospital Universitario de Burgos. Burgos, Spain |
| Ana Maria Arribas | Hospital Universitario de Burgos. Burgos, Spain |
| Jose Luis Teja | Hospital Universitario Marques de Valdecilla, Santander, Spain |
| Elsa Ots | Hospital Universitario Marques de Valdecilla, Santander, Spain |
| Juan Carlos Montejo | Hospital Universitario 12 de Octubre, Madrid, Spain |
| Mercedes Catalan | Hospital Universitario 12 de Octubre, Madrid, Spain |
| Isidro Prieto | Hospital Ramon y Cajal, Madrid, Spain |
| Gloria Gonzalo | Hospital Ramon y Cajal, Madrid, Spain |
| Beatriz Galvan | Hospital Universitario La Paz, Madrid, Spain |
| Miguel Angel Blasco | Hospital Universitario Severo Ochoa, Madrid, Spain |
| Estibaliz Meyer | Hospital Universitario Severo Ochoa, Madrid, Spain |
| Frutos Del Nogal | Hospital Universitario Severo Ochoa, Madrid, Spain |
| Loreto Vidaur | Hospital Universitario de Donostia, Donostia, Spain |
| Rosa Sebastian | Hospital Universitario de Donostia, Donostia, Spain |
| Pila Marco Garde | Hospital Universitario de Donostia, Donostia, Spain |
| Maria del Mar Martin Velasco | Hospital Universitario Nuestra Señora de Candelaria, Spain |
| Rafael Zaragoza Crespo | Hospital Universitario Dr. Peset, Spain |
| Mariano Esperatti | Institut Clínic del Tòrax, Hospital Clinic, IDIBAPS, Barcelona, Spain; Centro de Investigación Biomedica En  Red- Enfermedades Respiratorias (CibeRes). |
| Antoni Torres | Institut Clínic del Tòrax, Hospital Clinic, IDIBAPS, Barcelona, Spain; Centro de Investigación Biomedica En  Red- Enfermedades Respiratorias (CibeRes). |
| Philippe Montravers | Centre Hospitalier Universitaire Bichat-Claude Bernard, AP-HP, Université Paris VII, Paris, France |
| Olivier Baldesi | Centre Hospitalier Pays D Aix, Aix en Provence, France and AzuRea Group |
| Herve Dupont | Centre Hospitalier Universitaire d'Amiens, Amiens, France |
| Yazine Mahjoub | Centre Hospitalier-Universitaire d'Amiens, Amiens, France and AzuRea Group |
| Sigismond Lasocki | Centre Hospitalier-Universitaire d'Angers, Angers, France |
| Jean Michel Constantin | Centre Hospitalier Universitaire de Clermont-Ferrand, Clermont-Ferrand, France and AzuRea Group |
| Jean François Payen | Centre Hospitalier-Universitaire Grenoble, Grenoble France and AzuRea Group |
| Claude Martin | Hopital Nord, Marseille, France; AzuRea Group, France |
| Jacques Albanese | Hopital Nord, Marseille, France and AzuRea Group |
| Yannick Malledant | Hôpital Pontchaillou, Rennes, France |
| Julien Pottecher | University Hospital, Strasbourg, France and AzuRea Group |
| Jean-Yves Lefrant | Centre Hospitalier-Universitaire Nimes, Nimes France and AzuRea Group |
| Samir Jaber | Hospitalier-Universitaire Montpellier, Montpellier, France and AzuRea Group |
| Olivier Joannes-Boyau | Centre Hospitalier-Universitaire Bordeaux, Bordeaux, France and AzuRea Group |
| Christophe Orban | Centre Hospitalier-Universitaire Nice, Nice, France and AzuRea Group |
| Marlies Ostermann | St Thomas' Hospital, London, United Kingdom |
| Catherine McKenzie | St Thomas' Hospital, London, United Kingdom |
| Willaim Berry | St Thomas' Hospital, London, United Kingdom |
| John Smith | St Thomas' Hospital, London, United Kingdom |
| Katie Lei | St Thomas' Hospital, London, United Kingdom |
| Francesca Rubulotta | Charing Cross Hospital, Imperial Healthcare NHS Trust, London, United Kingdom |
| Anthony Gordon | Charing Cross Hospital, Imperial Healthcare NHS Trust, London, United Kingdom |
| Stephen Brett | Hammersmith Hospital, Imperial Healthcare NHS Trust, London, United Kingdom |
| Martin Stotz | St Mary's Hospital, Imperial Healthcare NHS Trust, London, United Kingdom |
| Maie Templeton | St Mary's Hospital, Imperial Healthcare NHS Trust, London, United Kingdom |
| Andrew Rhodes | St George's Hospital, St George's Healthcare NHS Trust, London, United Kingdom |
| Claudia Ebm | St George's Hospital, St George's Healthcare NHS Trust, London, United Kingdom |
| Carl Moran | St George's Hospital, St George's Healthcare NHS Trust, London, United Kingdom |
| Kirsi-Maija Kaukonen | Helsinki University Central Hospital, Helsinki, Finland |
| Ville Pettilä | Helsinki University Central Hospital, Helsinki, Finland |
| George Dimopoulos | Attikon University Hospital, Athens, Greece |
| Despoina Koulenti | Attikon University Hospital, Athens, Greece |
| Aglaia Xristodoulou | Attikon University Hospital, Athens, Greece |
| Vassiliki Theodorou | University Hospital of Alexandroupolis, Alexandroupolis, Greece |
| Georgios Kouliatsis | University Hospital of Alexandroupolis, Alexandroupolis, Greece |

| Stylianos Fotakis | University Hospital of Alexandroupolis, Alexandroupolis, Greece |
| --- | --- |
| Georgios Anthopoulos | 251 Air Force General Hospital of Athens, Athens, Greece |
| George Choutas | 251 Air Force General Hospital of Athens, Athens, Greece |
| Thanos Rantis | 251 Air Force General Hospital of Athens, Athens, Greece |
| Stylianos Karatzas | General Hospital of Athens ‘Hippokrateion’, Athens, Greece |
| Margarita Balla | General Hospital of Athens ‘Hippokrateion’, Athens, Greece |
| Metaxia Papanikolaou | General Hospital of Athens ‘Hippokrateion’, Athens, Greece |
| Pavlos Myrianthefs | ‘Aghioi Anargyroi’ Hospita, Athens, Greece |
| Alexandra Gavala | ‘Aghioi Anargyroi’ Hospita, Athens, Greece |
| Georgios Fildisis | ‘Aghioi Anargyroi’ Hospita, Athens, Greece |
| Antonia Koutsoukou | Sotiria General Hospital, Athens, Greece |
| Magdalini Kyriakopoulou | Sotiria General Hospital, Athens, Greece |
| Kalomoira Petrochilou | Sotiria General Hospital, Athens, Greece |
| Maria Kompoti | ‘Thriassio’ General Hospital of Eleusi, Athen, Greece |
| Martha Michalia | ‘Thriassio’ General Hospital of Eleusi, Athen, Greece |
| Fillis-Maria Clouva-Molyvdas | ‘Thriassio’ General Hospital of Eleusi, Athen, Greece |
| Georgios Gkiokas | Aretaieion University Hospital, Athens, Greece |
| Fotios Nikolakopoulos | Aretaieion University Hospital, Athens, Greece |
| Vasiliki Psychogiou | Aretaieion University Hospital, Athens, Greece |
| Polychronis Malliotakis | University Hospital Herakleion, Crete, Greece |
| Evangelia Akoumianaki | University Hospital Herakleion, Crete, Greece |
| Emmanouil Lilitsis | University Hospital Herakleion, Crete, Greece |
| Vassilios Koulouras | University Hospital of Ioannina, Ioannina, Greece |
| George Nakos | University Hospital of Ioannina, Ioannina, Greece |
| Mihalis Kalogirou | University Hospital of Ioannina, Ioannina, Greece |
| Apostolos Komnos | General Hospital of Larisa, Larisa, Greece |
| Tilemachos Zafeiridis | General Hospital of Larisa, Larisa, Greece |
| Achilleas Chovas | General Hospital of Larisa, Larisa, Greece |
| Kostoula Arvaniti | General Hospital of Thessaloniki ‘G. Papageorgiou’, Thessaloniki, Greece |
| Dimitrios Matamis | General Hospital of Thessaloniki ‘G. Papageorgiou’, Thessaloniki, Greece |
| Christos Chaintoutis | General Hospital of Thessaloniki ‘G. Papageorgiou’, Thessaloniki, Greece |
| Christina Kydona | General Hospital of Thessaloniki ‘Hippokrateion’, Thessaloniki, Greece |
| Nikoleta Gritsi-Gerogianni | General Hospital of Thessaloniki ‘Hippokrateion’, Thessaloniki, Greece |
| Tatiana Giasnetsova | General Hospital of Thessaloniki ‘Hippokrateion’, Thessaloniki, Greece |
| Maria Giannakou | Ahepa University Hospital, Thessaloniki, Greece |
| Ioanna Soultati | Ahepa University Hospital, Thessaloniki, Greece |
| Ilias chytas | Ahepa University Hospital, Thessaloniki, Greece |
| Eleni Antoniadou | General Hospital ‘G. Gennimatas’, Thessaloniki, Greece |
| Elli Antipa | General Hospital ‘G. Gennimatas’, Thessaloniki, Greece |
| Dimitrios Lathyris | General Hospital ‘G. Gennimatas’, Thessaloniki, Greece |
| Triantafyllia Koukoubani | General Hospital of Trikala, Trikala, Greece |
| Theoniki Paraforou | General Hospital of Trikala, Trikala, Greece |
| Kyriaki Spiropoulou | General Hospital of Trikala, Trikala, Greece |
| Vasileios Bekos | Naval Hospital of Athens, Athens, Greece |
| Anna Spring | Naval Hospital of Athens, Athens, Greece |
| Theodora Kalatzi | Naval Hospital of Athens, Athens, Greece |
| Hara Nikolaou | ‘Aghia Olga-Konstantopouleion’ General Hospital, Athens, Greece |
| Maria Laskou | ‘Aghia Olga-Konstantopouleion’ General Hospital, Athens, Greece |
| Ioannis Strouvalis | ‘Aghia Olga-Konstantopouleion’ General Hospital, Athens, Greece |
| Stavros Aloizos | General Hospital of Athens ‘NIMITS’, Athens, Greece |
| Spyridon Kapogiannis | General Hospital of Athens ‘NIMITS’, Athens, Greece |
| Ourania Soldatou | General Hospital of Athens ‘NIMITS’, Athens, Greece |
| Matteo Bassetti | Azienda Ospedaliera Universitaria Santa Maria della Misericordia, Udine, Italy |
| Chiara Adembri | Azienda Ospedaliero Universitaria Careggi, Florence, Italy |
| Gianluca Villa | Azienda Ospedaliero Universitaria Careggi, Florence, Italy |
| Antonio Giarratano | Università degli Studi di Palermo, Palermo, Italy |
| Santi Maurizio Raineri | Università degli Studi di Palermo, Palermo, Italy |
| Andrea Cortegiani | Università degli Studi di Palermo, Palermo, Italy |
| Francesca Montalto | Università degli Studi di Palermo, Palermo, Italy |
| Maria Teresa Strano | Università degli Studi di Palermo, Palermo, Italy |
| V. Marco Ranieri | San Giovanni-Battista Molinette, Turin, Italy |
| Claudio Sandroni | Catholic University School of Medicine, Rome, Italy |
| Gennaro De Pascale | Catholic University School of Medicine, Rome, Italy |
| Alexandre Molin | University of Genoa, Genoa, Italy |
| Paolo Pelosi | University of Genoa, Genoa, Italy |
| Luca Montagnani | Universitaria San Martino, Genova, Italy |
| Rosario Urbino | Universitaria S.Giovanni Battista della Città di Torino, Torino, Italy |
| Ilaria Mastromauro | Universitaria S.Giovanni Battista della Città di Torino, Torino, Italy |
| Francesco G. De Rosa | Universitaria S.Giovanni Battista della Città di Torino, Torino, Italy |
| V. Marco Ranieri | Universitaria S.Giovanni Battista della Città di Torino, Torino, Italy |
| Teresa Cardoso | Hospital de Santo António, Porto, Portugal |
| Susana Afonso | Hospital de St António dos Capuchos, Lisbon, Portgual |
| João Gonçalves-Pereira | Hospital de São Francisco Xavier, Lisbon, Portugal |
| João Pedro Baptista | Hospital de Universidade de Coimbra, Coimbra, Portugal |
| Murat Akova | Hacettepe University School of Medicine, Ankara, Turkey |
| Arife Özveren | Hacettepe University School of Medicine, Ankara, Turkey |
