# Supplementary material for: Does contemporary vancomycin dosing achieve therapeutic targets in a heterogeneous clinical cohort of critically ill patients? Data from the multinational DALI study
Source: Crit Care. 2014 May 15;18(3):R99. doi: 10.1186/cc13874 (PMC4075416; doi:10.1186/cc13874)
Supplement: Additional file 2 — A list of the contributing sites and their ethical approval bodies. [file cc13874-S2.docx]

**Appendix 2: Participating centres and approving ethics committee**

| **Country** | **City** | **Hospital** | **Ethics Committee*** |
| --- | --- | --- | --- |
| **Andorra** | Escaldes-Engordany | 1. Hospital Nostra Senyora de Meritxell | Hospital Nostra Senyora de Meritxell |
|  | | | |
| **Belgium** | Brussels | 1. St Luc Brussels | Commissie Voor Medische Ethiek, Universitair Ziekenhuis Gent |
|  | Brussels | 1. Universitair Ziekenhuis Brussels |  |
|  | Ghent | 1. Ghent University Hospital |  |
|  | Ottignies | 1. St Pierre |  |
|  | | | |
| **Great Britain** | London | 1. Imperial College: Charing Cross | National Research Ethics Service (NRES) London - Harrow |
|  | London | 1. Imperial College: Hammersmith |  |
|  | London | 1. Imperial College: St Marys Hospital |  |
|  | London | 1. St George's Hospital Medical School |  |
|  | | | |
| **Greece** | Athens | 1. Attikon University Hospital | Attikon University Hospital |
|  | Alexandroupolis | 1. University Hospital of Alexandroupolis Democritus | University Hospital of Alexandroupolis Democritus |
|  | Athens | 1. General Hospital of Athens ‘Hippokrateion’ | General Hospital of Athens ‘Hippokrateion’ |
|  | Athens | 1. ‘Sotiria’ General Hospital | ‘Sotiria’ General Hospital |
|  | Athens | 1. ‘Thriassio’ General Hospital of Eleusis | ‘Thriassio’ General Hospital of Eleusis |
|  | Athens | 1. ‘Aretaieion’ University Hospital | ‘Aretaieion’ University Hospital |
|  | Thessaloniki | 1. General Hospital of Thessaloniki ‘Hippokrateion’ | General Hospital of Thessaloniki ‘Hippokrateion’ |
|  | Athens | 1. ‘Aghia Olga-Konstantopouleion’ General Hospital | ‘Aghia Olga-Konstantopouleion’ General Hospital |
|  | | | |
| **Italy** | Bologna | 1. Azienda Ospedaliera Universitaria Palermo | Santa maria della Misericordia |
|  | Palermo | 1. Ospedale San Martino Genova |  |
|  | Turin | 1. San Giovanni-Battista Molinette |  |
|  | | | |
| **Portugal** | Porto | 1. Hospital de Santo António | Hospital de Santo António, Oporto Hospital Centre, Portugal |
|  | | | |
| **Spain**** | Barcelona | 1. Hospital Del Mar | Comunidad Autónoma de Catalunya |
|  | Las Palmas de Gran Canaria | 1. Hospital Universitario de Gran Canaria “Dr. Negrín” | Agencia Española del Medicamento y Producto Sanitario |
|  | Madrid | 1. Hospital Universitario 12 de Octubre |  |
|  | Teneriffe | 1. Hospital Universitario de Canarias | Agencia Española del Medicamento y Producto Sanitario |
|  | | | |
| **Turkey** | Ankara | 1. Hacettepe University | Hacettepe University |

*Additional approval from local institution review boards for which details have not been provided here

** All Spanish sites had a central Spanish Ethics Approval from *Agencia Española del Medicamento y Producto Sanitario* as well as from the Ethics Committee in the Spanish Region.
